# Will neoadjuvant chemoimmunotherapy complicate subsequent surgery compared with adjuvant chemoimmunotherapy?

**DOI:** 10.3389/fsurg.2026.1881043

**Published:** 2026-08-04

**Authors:** Zhoujunyi Tian, Haoshuai Yang, Jin Zhang, Zhenrong Zhang, Chaoyang Liang

**Affiliations:** Department of Thoracic Surgery, China-Japan Friendship Hospital, Beijing, China

**Keywords:** adjuvant therapy, inverse probability of treatment weighting (IPTW), neoadjuvant chemoimmunotherapy, non-small cell lung cancer, surgical difficulty

## Abstract

**Background:**

Neoadjuvant chemoimmunotherapy has significantly improved pathological response rates in resectable non-small cell lung cancer (NSCLC). However, whether it increases surgical complexity compared with upfront surgery followed by adjuvant therapy remains clinically debated.

**Methods:**

We retrospectively analyzed 121 NSCLC patients (81 neoadjuvant; 40 adjuvant) treated between 2021 and 2024. Inverse Probability of Treatment Weighting (IPTW) was applied to adjust for baseline imbalances. Surgical difficulty was quantified using a standardized 10-point scoring system. Multivariable logistic regression was then performed to identify independent risk factors for high-difficulty procedures (score >5).

**Results:**

IPTW successfully achieved balance across all baseline covariates. The neoadjuvant group exhibited significantly higher technical complexity than the adjuvant group (39.8% vs. 2.7% high difficulty; *P* < 0.001). Minimally invasive approach completion was substantially lower in the neoadjuvant group (57.8% vs. 93.5%), with significantly higher conversion rates (33.9% vs. 6.5%; *P* < 0.001). Anatomical challenges, particularly dense hilar adhesions and vascular fibrosis (the “frozen hilum”), were more prevalent after neoadjuvant treatment (*P* < 0.05). Multivariable analysis confirmed neoadjuvant therapy as the primary independent driver of increased surgical difficulty (OR: 0.032 for adjuvant vs. neoadjuvant; *P* < 0.001). Postoperative recovery metrics, including chest tube duration and hospital stay, remained comparable between groups after weighting (*P* > 0.05).

**Conclusion:**

Neoadjuvant chemoimmunotherapy was associated with greater technical surgical difficulty and higher conversion rates compared with upfront surgery followed by adjuvant therapy. Despite these technical challenges, surgical resection remains safe and feasible, though it requires specialized thoracic expertise to manage complex anatomical alterations effectively.

## Introduction

Lung cancer is the leading cause of cancer-related mortality worldwide (1). Non-small cell lung cancer (NSCLC) accounts for about 85% of all lung cancers (2). 20% to 30% of patients have resectable advanced lung cancer at diagnosis. However, prognosis after surgery for these patients is poor (3, 4). Neoadjuvant or adjuvant chemotherapy has shown limited improvement in survival, but the addition of immunotherapy brings hope. Checkmate-816, Neotorch, AEGEAN, and KENOTE-671 trials reported that neoadjuvant chemoimmunotherapy (NCI) improved event-free survival (EFS) and pathological complete regression (pCR) compared with chemotherapy alone (5–10). Long-term outcome of KEYNOTE −671 indicated the significant overall survival benefit of neoadjuvant pembrolizumab plus chemotherapy followed by adjuvant pembrolizumab compared with neoadjuvant chemotherapy alone, and showed that EFS benefit of this scheme could bring OS benefit (11). These trials have led to a new paradigm in the multidisplinary management of resectable NSCLC.

Surgery remains an integral part of resectable lung cancer, and immunotherapy has also gained an important place in multidisciplinary therapy in recent years. Extensive clinical application of immunotherapy for resectable NSCLC raises questions about timing of immunotherapy intervention and their impact on surgery. Previous large sample studies showed that the additional complications and mortality caused by surgery after neoadjuvant chemotherapy or neoadjuvant chemoradiotherapy, even complex lung resection, are controllable (12). Whether the addition of immunotherapy in neoadjuvant therapy has an impact on surgery is still controversial. There is little research on the specific technical aspects of surgery after neoadjuvant immunotherapy. Data from SAKK 16/14 and NADIM trials indicated that conversion rate was lower than 20%, R0 resection rate was higher than 90%, and a relatively low surgery cancellation rate of 18% due to tumor progression or drug toxicity (13, 14). A systematic review concluded surgical outcomes and perioperative complications after neoadjuvant immunotherapy were acceptable (15). On the contrary, neoadjuvant chemoimmunotherapy may cause inflammation, fibrosis or adhesion of the tissue around the tumor and lymph nodes, resulting in blurred anatomical structure and increasing the difficulty of surgical separation and excision. In addition, immunotherapy may cause immune-related adverse reactions such as pneumonia, myocarditis, further increasing the perioperative risk. Several studies suggested that the toxicity of neoadjuvant immunotherapy and technical difficulty of surgery may increase the risk of perioperative complications. 15%–63% did not undergo surgery due to grade 3–4 adverse effects caused by toxicity, and 16%–22% did not undergo surgery due to disease progression (7–10). 10% to 15% delayed surgery with a median delay of 2 weeks (7). A study evaluated technical challenges encountered during resection after neoadjuvant therapy and reported that the conversion rate of stage I, IIA and IIB-IIIA was 25%, 50% and 71% respectively, mainly due to inflammation, severe adhesion and fibrosis in the pulmonary fissure, hilar and mediastinal lymph nodes (16). Therefore, some researchers suggest that surgery followed by adjuvant immunotherapy should be considered for patients with resectable lung cancer (17).

Recent phase III trials did not directly compare neoadjuvant and adjuvant chemoimmunotherapy with respect to surgical complexity. There is therefore a need to quantify whether neoadjuvant chemoimmunotherapy is associated with greater operative difficulty and whether this translates into worse short-term recovery compared with upfront surgery followed by adjuvant chemoimmunotherapy (ACI). This study addresses this critical gap by comparing detailed metrics of surgical difficulty and objective perioperative outcomes between patients receiving NCI followed by surgery (NT group) and those receiving upfront surgery followed by ACI (AT group). Given the non-randomized retrospective design, the present study used IPTW to reduce measured baseline imbalances and to estimate associations between treatment sequence and surgical outcomes.

## Methods

### Patient selection

Patients with resectable NSCLC at AJCC 8th stage IIA to IIIB received multidisciplinary therapy in China-Japan Friendship Hospital between June 2019 and June 2024 were reviewed. Patients received neoadjuvant chemoimmunotherapy followed by surgery or upfront surgery followed by adjuvant immunotherapy with or without chemotherapy were included. The exclusion criteria were: (1) patients aged <18 or >75; (2) patients receiving neoadjuvant immunotherapy alone or neoadjuvant chemotherapy alone; (3) the objective of surgery is not radical such as biopsy, wedge resection or segment resection. According to the sequential relationship between immunotherapy and surgery, included patients were divided into neoadjuvant therapy group (NT group) and adjuvant therapy group (AT group). Postoperative adjuvant therapy was not prescribed for patients in the neoadjuvant therapy group. This retrospective study was approved by the Institutional Review Board of China-japan Friendship Hospital (ID: 2023-KY-151, date: July 5, 2023). Informed consent of patients was waived given the retrospective setting.

### Data collection

The following data were collected: patients baseline characteristic including age, gender, smoke history, pulmonary function, comorbidity, location of disease, pathological subtype, and stage; surgical outcomes including surgery approach, procedure, operation duration, intraoperative blood loss, R0 resection rate, drainage volume, drainage duration and postoperative hospital stay; surgery difficulty; and postoperative complications; and for NT group patients, the interval between completion of neoadjuvant chemoimmunotherapy and surgery. Surgical approach was categorized as video-assisted thoracoscopic surgery (VATS), conversion from VATS to thoracotomy, or planned thoracotomy. Robotic-assisted thoracic surgery was not performed in this cohort; therefore, all minimally invasive procedures in the analysis were VATS procedures.

### Surgical difficulty assessment

Surgical difficulty was evaluated using a structured 10-point operative difficulty score established based on previous research (18, 22). The score was assessed according to the following five indicators. Severe pleural adhesion refers to adhesion of the visceral pleura to the chest wall that requires sharp separation of energy instruments and takes more than half an hour. Lymph node enlargement refers to the short diameter of lymph nodes increased to more than 1 cm. Adhesion between lymph node and vessels means that the adhesion makes it difficult to dissect blood vessels such as arteries or veins. Vascular sheath difficulty refers to the dissection of the vascular sheath is difficult due to the reaction after treatment. Peribronchial adhesion refers to the difficulty of dissecting the bronchus due to adhesion or lymph node enlargement caused by the surrounding bronchial structure after treatment. Those changes above might be related to a diffuse fibrotic reaction, tissue edema, lack of interstitial space, and compromised vascularization caused by preoperative chemoimmunotherapy (19). Each indicator had three ratings based on degree (0 = none/easy; 1 = mild/middle/single; 2 = hard/severe/multiple). Surgical difficulty score was the sum of the five scores, with a value between 0 and 10. A score >5 was defined as high surgical difficulty.

To improve reproducibility, operative records were reviewed independently by two thoracic surgeons. Disagreements were resolved by consultation with a third senior thoracic surgeon until consensus was reached.

### Inverse probability of treatment weighting (IPTW) and model construction

To reduce confounding from baseline differences between treatment groups, IPTW was applied using propensity scores estimated from clinically relevant pretreatment covariates, including age, sex, smoking history, COPD status, clinical T/N stage, and pathological subtype. IPTW creates a weighted pseudo-population in which measured covariates are more comparable between groups, but it cannot eliminate residual confounding from unmeasured or incompletely balanced factors.

The quality of balance achieved after IPTW was assessed using the Standardized Mean Difference (SMD), with a threshold of SMD < 0.1 traditionally indicating adequate balance. Comparisons of continuous outcomes in the IPTW-weighted cohort were performed using the weighted t-test or weighted Wilcoxon test, while categorical variables employed the weighted X^2^ test.

Finally, univariate logistic regression was conducted on the weighted patient cohort to identify independent factors associated with a high surgical difficulty outcome. Variables demonstrating a statistical association (*p* < 0.05) in the univariate models were considered for inclusion in a multivariate model.

### Statistical analyses

Baseline characteristics were compared between two groups. Categorical variables were compared using Pearson's *Χ*2 or Fisher's exact test. Continuous variables that behaved as normally distributed were evaluated using the Student's t test, while variables that did not behave as normally distributed were compared using the Mann–Whitney U test. Univariate and multivariate binary logistics regressions were conducted to adjust the effects of chosen variables and identify factors associated with surgery difficulty. Variables with *p* < 0.05 in the univariate regression analyses were included in multivariate regression analyses.

A two-sided *P* value < 0.05 was considered statistically significant. All statistical analyses were performed using Stata/SE version 14.0 for Windows (StataCorp, College Station, TX, USA). PSM was performed by R for Windows version 4.3.2 [R Core Team (2023). R: A Language and Environment for Statistical Computing. R Foundation for Statistical Computing, Vienna, Austria. https://www.R-project.org/].

## Results

### Patient enrollment and selection

**Figure 1:**
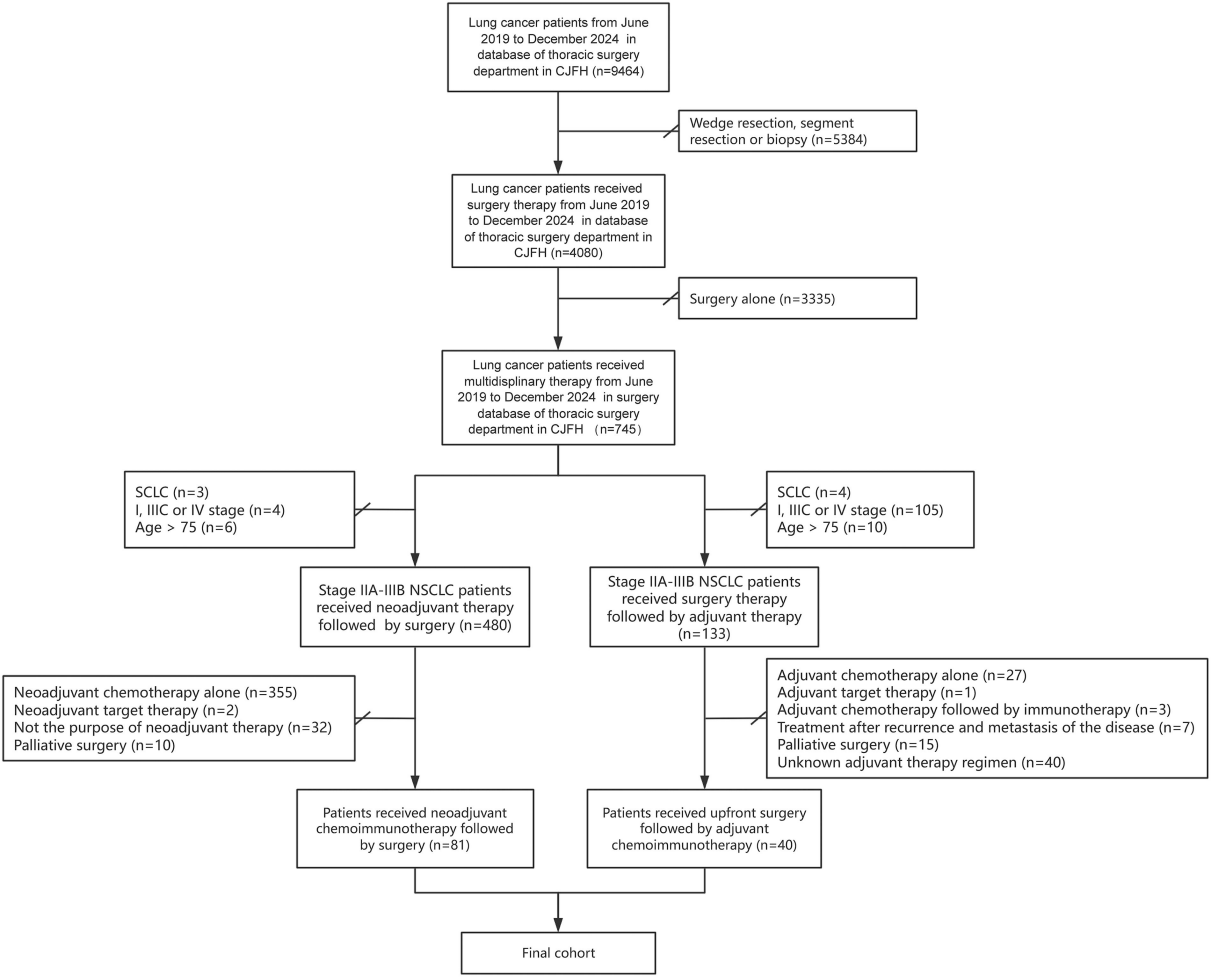
Flowchart of selection of study cohort. CJFH, China-Japan friendship hospital; SCLC, small cell lung cancer; NSCLC, non-small cell lung cancer.

A total of 121 patients with resectable non-small cell lung cancer (NSCLC) at AJCC 8th stage IIA–IIIB were included in this study (Figure 1). Among them, 81 patients received neoadjuvant chemoimmunotherapy followed by surgery (NT group), while 40 patients underwent upfront surgery followed by adjuvant chemoimmunotherapy (AT group).

Before matching, significant differences were observed between the two groups. Patients in the NT group were older (mean age: 63.2 vs. 59.8 years, *P* = 0.018), had a higher proportion of males (92.6% vs. 70.0%, *P* = 0.001), and a greater prevalence of smoking history (87.6% vs. 55.0%, *P* < 0.001). Pathologically, squamous carcinoma was more common in the NT group (82.0% vs. 22.5%, *P* < 0.001), while adenocarcinoma was more frequent in the AT group (72.5% vs. 16.7%, *P* < 0.001). 39 of 81(48.1%) patients in neoadjuvant group achieved pCR. In the NT group, the median interval between completion of NCI and surgery was 34 days (Q1-Q3: 28–41 days).

After IPTW, the weighted sample sizes were *n* = 108.8 for the NT group and *n* = 107.7 for the AT group. The IPTW procedure demonstrated successful stabilization and substantial improvement in balance across key clinical and procedural variables, achieving negligible standardized mean differences (SMDs) or large *P*-values, indicating that the pseudo-population was adequately matched for the comparison (Table 1). Age showed the most dramatic improvement, with its substantial initial imbalance (SMD 0.445) stabilizing at a well-balanced 0.122 (*P* = 0.712). Although FEV1% still had a relatively high post-weighting SMD (0.775), the weighted statistical test indicated no significant difference between the groups (*P* = 0.112), validating the weighted cohort's utility for this critical physiological metric.

**Table 1:** Patient characteristics between neoadjuvant chemoimmunotherapy group and adjuvant chemoimmunotherapy group before and after matching.

Variables	All inluded patients				IPTW-weighted patients			
	NT group (*n* = 81)	AT group (*n* = 40)	P	SMD	NT group (*n* = 109)	AT group (*n* = 108)	P	SMD
Age, mean ± SD	63.2 ± 6.8	59.8 ± 8.8	**0** **.** **03**	**0**.**44**	63.2 ± 6.4	64.1 ± 8.1	0.71	0.12
Gender, n(%)			**0.003**				1	
Male	75 (92.6)	28 (70.0)			95 (87.0)	93 (86.3)		
Female	6 (7.4)	12 (30.0)			14 (13.0)	15 (13.7)		
Smoking history, n(%)	71 (87.7)	22 (55.0)	**<0**.**001**		86 (79.1)	81 (75.4)	0.64	
Pathological Subtype			**<0.001**				0.40	
Adenocarcinoma	13 (16.0)	29 (72.5)			30 (27.5)	38 (35.0)		
Squamous carcinoma	67 (82.7)	9 (22.5)			78 (71.3)	68 (62.9)		
Large cell carcinoma	1 (1.3)	2 (5.0)			1 (1.1)	2 (2.0)		
Pulmonary function								
FEV1/FVC(%), mean ± SD	70.7 ± 10.5	73.8 ± 7.0	0.21	0.34	71.1 ± 10.1	76.5 ± 6.0	0.16	0.6
FEV1%, mean ± SD	89.8 ± 15.8	103.9 ± 15.8	**0**.**006**	**0**.**89**	92.0 ± 15.7	102.9 ± 12.4	0.11	0.78
DLCO SB%, mean ± SD	76.1 ± 17.3	83.4 ± 23.9	0.36	0.35	75.1 ± 10.1	76.1 ± 6.0	0.29	0.65
Comorbidity, n(%)								
Hypertension	41 (50.6)	21 (52.5)	0.99		49 (45.4)	67 (62.6)	**0**.**016**	
Diabetes	15 (18.5)	10 (25.0)	0.56		18 (16.3)	45 (42.1)	**<0**.**001**	
Cardiac disease	9 (11.1)	1 (2.5)	0.16		10 (9.5)	1 (1.5)	**0**.**02**	
COPD	37 (45.7)	5 (12.5)	**<0**.**001**		10 (52.6)	3 (15.8)	**0**.**019**	
Other	8 (9.9)	5 (12.5)	0.89		20 (18.5)	21 (19.3)	1	
Lobe, n(%)			0.44				0.55	
Right upper	18 (22.2)	7 (17.5)			21 (19.0)	24 (22.1)		
Right middle	4 (4.9)	1 (2.5)			4 (4.1)	2 (1.6)		
Right lower	17 (21.0)	15 (37.5)			26 (23.9)	33 (30.6)		
Left upper	22 (27.2)	8 (20.0)			25 (23.2)	20 (18.6)		
Left lower	20 (24.7)	9 (22.5)			32 (29.8)	29 (27.1)		
AJCC 7th T stage, n(%)			**0.02**				0.15	
T1	10 (12.3)	13 (32.5)			15 (14.0)	17 (15.6)		
T2	40 (49.4)	20 (50.0)			55 (50.9)	45 (41.5)		
T3	19 (23.5)	3 (7.5)			23 (21.1)	36 (33.7)		
T4	12 (14.8)	4 (10.0)			15 (14.0)	10 (9.1)		
AJCC 7th N stage, n(%)			0.98				0.66	
N0	33 (40.7)	17 (42.5)			45 (41.7)	51 (47.7)		
N1	16 (19.8)	8(20.0)			21 (19.7)	20 (18.7)		
N2	32(39.5)	15(37.5)			42 (38.6)	36 (33.7)		

*P* values in bold are statistically significant. IPTW, inverse probability of treatment weighting; SMD, standardized mean differences; NT group, neoadjuvant chemoimmunotherapy therapy group; AT group, adjuvant chemoimmunotherapy therapy group; DLCO SB, diffusing capacity of the lung for carbon monoxide, single-breath method %. Its percentage of prediction value, FVC, forced-vital capacity; FEV1, forced expiratory volume in 1 s, FEV1/FVC (%). The percentage calculated by dividing FEV1 by FVC. SD, standard deviation; COPD, chronic obstructive pulmonary disease; AJCC, American joint committee on cancer.

### Surgical outcomes and difficulty assessment

Surgical outcomes were shown in Table 2. The surgical approach was sharply impacted by the timing of treatment. The distribution of approaches (VATS, Conversion, Direct thoracotomy) was significantly different (*P* < 0.001). The NT group utilized a minimally invasive approach in only 57.8% of cases, substantially lower than the 93.5% seen in the AT group. This difference led to a significantly higher conversion rate to open surgery in the NT group (33.9% vs. 6.5%) and a small percentage of cases requiring direct open surgery (8.3% vs. 0%). Perhaps the higher proportion of complex surgical procedures in the NT group also contributed to part of the difference. There were statistically significant differences in the bleeding volume during the surgery and drainage after surgery, but the clinical significances were not substantial.

**Table 2:** Surgical outcomes of weighted patients.

Variables	NT group (*n* = 109)	AT group (*n* = 108)	P
Surgery approach			**<0** **.** **001**
VATS	63 (57.8)	101 (93.5)	
Conversion	37 (33.9)	7 (6.5)	
Thoracotomy	9 (8.3)	0 (0)	
Operation procedure			**0**.**003**
Lobectomy, n(%)	96 (88.1)	108 (100)	
Bilobectomy, n(%)	4 (3.7)	0	
Sleeve Lobectomy, n(%)	4 (3.7)	0	
Pneumonectomy, n(%)	5 (4.6)	0	
Operation duration(min), median(Q1,Q3)	160 (130,194)	137.5 (110,182)	0.30
Bleeding volume(mL), median(Q1,Q3)	50 (20,100)	20 (20,50)	**0**.**03**
Resection completeness			0.39
R0	107 (97.9)	108 (100)	
R1/2	2 (2.1)	0 (0)	
Chest tube duration(days), median(Q1,Q3)	4 (3,6)	4 (3,5)	1
Postoperative hospital duration(days), median(Q1,Q3)	6 (5,8)	5 (4,6)	0.14
First day drainage volume(mL), mean ± SD	295 ± 152	197 ± 125	**0**.**02**

*P* values in bold are statistically significant. NT group, neoadjuvant chemoimmunotherapy therapy group; AT group, adjuvant chemoimmunotherapy therapy group; VATS, video-assisted thoracoscopic surgery; Q1, first percentile; Q3, third percentile; SD, standard deviation.

The weighted cohort analysis revealed that neoadjuvant therapy group significantly increased the technical complexity and difficulty of subsequent surgical procedures compared to patients receiving adjuvant therapy group. The NT group resulted in a significantly higher proportion of high-difficulty surgery (scoring >5), accounting for 39.8% of cases, vs. only 2.7% in the AT group. The assessment of surgical difficulty demonstrated that patients in the NT group faced greater challenges during surgery. Adhesion between lymph nodes and vessels was more common in the NT group (52.6% vs. 21.1%, *P* = 0.043), as was the difficulty in dissecting the vascular sheath (52.6% vs. 15.8%, *P* = 0.044). Severe pleural adhesions (26.3% vs. 15.8%) and multiple lymph node enlargement (42.1% vs. 10.5%) were also more frequently observed in the NT group, although these differences did not reach statistical significance (Table 3).

**Table 3:** Surgery difficulty of weighted patients.

Variables	NT group (*n* = 109)	AT group (*n* = 108)	P
Pleural adhesion			0.51
None	39 (36%)	47 (43.2%)	
Mild	47 (43.1%)	39 (36.2%)	
Severe	23 (21%)	22 (20.6%)	
Lymph node enlargement			**<0** **.** **001**
None	51 (47.1%)	77 (72%)	
Single	19 (17.2%)	7 (7%)	
Multiple	39 (35.7%)	23 (21.1%)	
LN-vessels adhesion			**<0**.**001**
None	48 (43.7%)	74 (69.2%)	
Mild	44 (40%)	32 (29.8%)	
Severe	18 (16.3%)	1 (1.1%)	
Vascular sheath difficulty			**<0**.**001**
Easy	45 (41.6%)	80 (74.3%)	
Middle	35 (32.4%)	25 (23.1%)	
Hard	28 (25.9%)	3 (2.7%)	
Peribronchial adhesion			**<0**.**001**
Easy	50 (46.7%)	96 (89.2%)	
Middle	35 (32.8%)	12 (10.8%)	
Hard	22 (20.5%)	0 (0%)	

*P* values in bold are statistically significant. NT group, neoadjuvant chemoimmunotherapy therapy group; AT group, adjuvant chemoimmunotherapy therapy group; LN, lymph node.

### Postoperative complications

The IPTW-weighted comparative analysis was essential for determining if the documented increase in surgical difficulty translated into clinically meaningful adverse outcomes. The analysis revealed objective, quantitative differences in immediate technical consequences related to dissection. There were differences in postoperative complications between the two groups. The incidence of postoperative pulmonary infection (22.5% vs. 9.4%, *P* = 0.01) and atrial fibrillation (10.9% vs. 1.5%, *P* = 0.010) in the NT group was significantly higher than that in the AT group (Table 4).

**Table 4:** Postoperative complications of weighted patients.

Variables	NT group (*n* = 109)	AT group (*n* = 108)	P
Postoperative complication			
Pneumothorax	5 (4.9%)	3 (2.5%)	0.56
Hemothorax	1 (0.9%)	0 (0%)	0.99
Subcutaneous emphysema	1 (1.1%)	4 (3.9%)	0.38
Pulmonary infection	25 (22.5%)	10 (9.4%)	**0** **.** **01**
Pulmonary atelectasis	4 (3.3%)	2 (2.1%)	0.91
Pulmonary embolism	1 (0.9%)	4 (4%)	0.31
Prolonged air leak	2 (2%)	0 (0%)	0.41
Pleural effusion	8 (7.1%)	6 (5.5%)	0.84
Atrial fibrillation	12 (10.9%)	2 (1.5%)	**0**.**01**
Heart failure	0 (0%)	3 (3%)	0.21
Cerebral infarction	3 (2.6%)	0 (0%)	0.27

*P* values in bold are statistically significant. NT group, neoadjuvant chemoimmunotherapy therapy group; AT group, adjuvant chemoimmunotherapy therapy group.

However, when evaluating some other key indicators of clinical recovery, no statistically significant differences were observed between the NT and AT groups in the weighted population. Operative duration did not differ significantly (median: 160 min vs. 137.5 min, *P* = 0.30), nor did chest tube duration (median: 4 days vs. 4 days, *P* = 1), postoperative hospital stay (median: 6 days vs. 5 days, *P* = 0.14), or overall rates of postoperative complication (Table 2).

### Risk factors for high surgical difficulty

To formally identify independent risk factors for high surgical difficulty group, a weighted multivariate logistic regression model was constructed using IPTW-adjusted data (Table 5). The model identifies the NT group as the only independent predictor of high surgical difficulty (*P* < 0.001). Compared to the NT group, assignment to the AT arm is associated with a significant reduction in the odds of technical complexity (OR: 0.03; 95% CI: 0.01–0.21). Other adjusted factors, including sex (*P* = 0.10), clinical T stage (*P* > 0.05), and COPD (*P* = 0.49), did not reach statistical significance in the final multivariate model. With a McFadden's pseudo R^2^ of 0.322 indicating a robust fit, the results confirm that the treatment sequence itself—specifically the use of neoadjuvant chemoimmunotherapy—is the principal independent driver of increased operative difficulty, rather than pre-existing patient conditions.

**Table 5:** IPTW-adjusted univariate and multivariate binary logistics regressions analyses for factors of surgery difficulty score >5.

Variable	Univariate regression			Multivariate regression		
	OR	95%CI	P	OR	95%CI	P
Age	0.96	0.91–1.02	0.22			
Gender						
male	Ref			Ref		
female	0.13	0.02–1.11	0.06	0.14	0.01–1.45	0.10
Smoking history	1.62	0.35–7.57	0.54			
Pathological Subtype						
Squamous carcinoma	Ref					
Adenocarcinoma	0.91	0.27–3.05	0.88			
Large cell carcinoma	2.02	0.17–24.83	0.58			
Pulmonary function						
FEV1/FVC(%)	1.00	0.93–1.09	0.91			
FEV1%	1.00	0.96–1.05	0.99			
DLCO SB%	1.00	0.96–1.05	0.97			
Comorbidity						
Hypertension	0.63	0.21–1.89	0.41			
Diabetes	0.62	0.16–2.39	0.49			
Cardiac disease	0.83	0.16–4.42	0.83			
COPD	2.64	0.88–7.92	0.09	1.54	0.46–5.17	0.49
Other	0.69	0.11–4.36	0.69			
Lobe						
Right upper	Ref					
Right middle	2.01	0.15–26.31	0.60			
Right lower	3.26	0.63–16.92	0.16			
Left upper	2.37	0.46–12.16	0.30			
Left lower	2.14	0.37–12.30	0.40			
AJCC 8th T stage						
T1	Ref			Ref		
T2	0.37	0.10–1.31	0.13	0.18	0.03–1.12	0.07
T3	0.27	0.05–1.31	0.11	0.22	0.03–1.54	0.13
T4	0.27	0.04–1.93	0.19	0.11	0.01–1.26	0.08
AJCC 8th N stage						
N0	Ref					
N1	2.27	0.52–9.82	0.28			
N2	1.88	0.56–6.34	0.31			
Neoadjuvant or adjuvant therapy						
Neoadjuvant	Ref			Ref		
Adjuvant	0.04	0.01–0.35	**0** **.** **004**	0.03	0.01–0.21	**<0**.**001**

*P* values in bold are statistically significant. IPTW, inverse probability of treatment weighting; CI, confidence interval; OR, odds ratio.

## Disscussion

Neoadjuvant chemoimmunotherapy has become an important treatment strategy for resectable NSCLC because of its favorable pathological response and survival outcomes (7, 11). The influence of neoadjuvant chemoimmunotherapy on the surgery technical difficulty, surgery delay and cancellation remains controversial. In this retrospective analyses involving resectable NSCLC patients who receive neoadjuvant chemoimmunotherapy before sequent radical excision, or upfront surgery before adjuvant chemoimmunotherapy, we compared the perioperative outcomes and surgery difficulty between the two groups. In this retrospective cohort, neoadjuvant chemoimmunotherapy was associated with greater surgical difficulty, mainly due to increased lymph node-vessel adhesion, vascular sheath difficulty, and peribronchial adhesion, which resulted in increased conversion rate, intraoperative blood loss and first 24-hour drainage volume after surgery. Even the postoperative complications in the neoadjuvant group were worse than that in the adjuvant group before and after IPTW matching, no significant difference was observed in the incidence of postoperative complications.

Xu and colleagues reported that neoadjuvant immunotherapy resulted in a 87% radical resection rate, no surgery delay and 21.7% complication rate in 23 patients with squamous carcinoma clinically staged at IIB to IIIC (20). Hong and colleagues assessed 25 NSCLC patients with clinical stage IIA-IIIC and reported a 100% R0 resection rate and a 52% overall postoperative complication rate (21). The above study has some problems, such as no control group and small sample size. A relatively high R0 resection and low postoperative complication rate was achieved in our study, and no significant difference was detected compared with adjuvant therapy group. Zhang and colleagues compared 37 patients at clinical stage IA to IIIB who received neoadjuvant immunotherapy with patients who received upfront surgery or neoadjuvant chemotherapy, and reported a higher postoperative complication rate of 37.8% but without increase of surgery difficulty (22). There are some differences between their results and the present study. They found the lymph node dissection was more difficult in neoadjuvant immunotherapy group but intrathoracic adhesion, tumor invasion, and whole procedure diffculty were similar compared to other group, while it leads to a higher incidence of postoperative complications. We consider that this may be due to the presence of early stage lung cancer patients in the control group, whose general status is significantly better than that of patients requiring neoadjuvant immunotherapy.

The significant increase in the conversion rate of thoracotomy and the difficulty of surgery suggested the influence of neoadjuvant immunotherapy on surgery. However, there were no significant differences in operation duration, intraoperative blood loss, postoperative drainage volume, postoperative chest tube duration, postoperative hospital stay and postoperative complication rate, indicating that the effect of neoadjuvant therapy on surgery did not bring substantial changes in perioperative outcomes. Other less frequent but serious complications such as 30-day mortality and fistula were not reported in the study. A systematic review including 18 studies reported a 30-day mortality ranged from 0 to 5.4%, with 11 trials (61.1%) reporting 0%, and few studies reported fistula rate from 0 to 25% (15). The limitation of sample size may have limited the number of perioperative events, resulting in no significant difference in complication rate between the two groups. The apparent discrepancy between increased pulmonary infection, atrial fibrillation, and conversion to thoracotomy on one hand, and similar chest tube duration and postoperative hospital stay on the other, requires careful interpretation. We do not consider the two strategies to be equally safe in every respect. Rather, our data suggest that, in selected patients managed at an experienced thoracic center, the additional technical complexity did not clearly prolong several short-term recovery indicators, while it did increase specific perioperative risks. This distinction is important for preoperative counseling, operating-room planning, pulmonary infection prevention, and postoperative rhythm monitoring. The higher pulmonary infection rate may reflect greater tissue trauma, more extensive dissection, conversion to thoracotomy, treatment-related immune or inflammatory changes, or differences in baseline pulmonary reserve. The higher atrial fibrillation rate may similarly be related to greater hilar manipulation, inflammation, and surgical stress. These findings support the need for careful patient selection, experienced surgical teams, and structured postoperative monitoring after neoadjuvant chemoimmunotherapy.

The study of patient selection and the precise definition of “resectable” will help to increase the benefit of patients receiving neoadjuvant immunotherapy plus surgery. Using a clear and uniform standard when defining resectability would help identify suitable patients for multidisplinary therapy. The International Association for the Study of Lung Cancer reported a definition of “resectable” included IIIA (N0–1), partial N2 with single-station mediastinal lymph node metastasis and the short diameter of lymph node <2 cm, partial T4 (satellite nodules in the adjacent lobe) N1; potential resectable includes partial stage IIIA and IIIB with the short diameter of single-station N2 mediastinal lymph node < 3 cm, other potentially resectable T3 or T4 central tumor (23). Several efforts have been made but not yet resulted in a consensus that is consistent and convenient for clinical practice (24). For early stage patients such as lymph node non-metastasis or non-N2 patients, upfront surgery combined with adjuvant immunotherapy is more conducive to complete resection and accurate staging and reduce the risk of surgical complications. Postoperative adjuvant immunotherapy rather than preoperative immunotherapy could help avoid treatment-related adverse effects affecting the continuity of treatment (17).

This study has several limitations. First, this was a retrospective analysis and the small sample size will increase the risk of type II error. Second, treatment assignment was not randomized, and the two groups differed substantially at baseline. Although IPTW was applied, residual confounding from unmeasured variables, incomplete balance, surgical selection, tumor centrality, treatment regimen, and surgeon experience may remain. Therefore, causal conclusions should be avoided. Third, it was a real-world study but single-center experience; thus, the findings may not be generalized to other institutions. Forth, the surgical difficulty score was locally developed and has not been externally validated. Future studies should externally validate the score and report inter-rater agreement. Finally, the interval between completion of neoadjuvant therapy and surgery may influence inflammatory or fibrotic changes and should be evaluated in larger datasets. Therefore our results should be interpreted with caution.

## Conclusion

Neoadjuvant chemoimmunotherapy was associated with a lower rate of minimally invasive completion and a higher level of surgical difficulty compared with upfront surgery followed by adjuvant therapy. In selected patients treated at an experienced thoracic center, these technical challenges did not significantly prolong chest tube duration or postoperative hospital stay, but higher rates of pulmonary infection, atrial fibrillation, and conversion to thoracotomy indicate increased perioperative risk. Prospective multicenter studies are required to validate these findings and to define the optimal treatment sequence for different surgical-risk subgroups.

## Data Availability

Due to privacy and ethical restrictions, requests to access the raw data should be directed to the corresponding author.
